# In vitro antibacterial activity of fruit pulp extracts of *Tamarindus indica* against *Staphylococcus aureus* and *Klebsiella pneumoniae*

**DOI:** 10.1186/s12906-024-04404-6

**Published:** 2024-03-19

**Authors:** Gatluak Goanar, Geremew Tafesse, Workineh Mengesha Fereja

**Affiliations:** 1 Department of Biology, Dilla University, Dilla, Ethiopia; 2 Department of Chemistry, and Energy and Environment Research Center, Dilla University, Dilla, Ethiopia

**Keywords:** Antibacterial activity, Disk diffusion, MIC, Pathogenic bacteria, *Tamarindus indica*

## Abstract

**Background:**

Infectious diseases are increasingly recognized as public health concern worldwide as the rising incidence in multidrug resistance bacteria. This consequently enforces the need to find a new antimicrobial agent where plants have a potential source. This study investigated the antibacterial activity of fruit pulp extract of the *Tamarindus indica* against *Staphylococcus aureus* (*S. aureus*) and *Klebsiella pneumoniae* (*K. pneumoniae*).

**Methods and materials:**

Maceration technique was employed for subsequent extraction of the sample using acetone and ethanol. Antibacterial activity of the plant extract was investigated based on minimum inhibitory concentration (MIC) against Gram-negative strain (*K. pneumoniae* (ATCC 700603)) and Gram-positive strain (*S. aureus* (ATCC 25923)) using agar disc-diffusion technique.

**Results:**

It was found that both acetone and ethanol extracts showed significant antibacterial activities, against both *S. aureus* and *K. pneumoniae* as compared to the negative control (*P* = 0.00), but no significantly different from the drug (*P *> 0.05). However, *K. pneumoniae* showed more sensitivity to the extracts than *S. aureus* with MIC value of 18.75 mg/mL and 9.38 mg/mL for both acetone and ethanol extracts against *S. aureus* and *K. pneumoniae* respectively.

**Conclusion:**

This study suggested that the fruit pulp have antibacterial properties, which might validate their traditional uses.

## Introduction

Infectious diseases are increasingly recognized as global public health concern in consonance with rising incidence in multidrug resistance of pathogenic bacteria [1, 2]. In addition, the high cost, negative side effects associated with and the inaccessibility of the synthetic antimicrobial agents especially for rural populations in developing countries are the major challenge in global health care [3]. Against this backdrop, the development of alternative drug to treat such infectious diseases is urgently required [3]. This further imposes researchers to discover new antimicrobial agents are exclusively important [4–7].

Recently, researchers have shown an increasing interest in herbal medicines because of their relative safety and tolerability as compared to modern medicines [8]. Moreover, plants have an amazing ability to produce different phytochemicals in the secondary phase of plants metabolism, including alkaloids, glycosides, terpenoids, saponins, steroids, flavonoids, tannins, quinones and coumarins [1, 3]. These secondary metabolites are responsible in the treatment of bacterial infections [9, 10] which make them valuable in traditional medicine for the treatment of chronic and common microbial infections [11, 12]. Some natural products are highly effective to combat bacterial resistance of with high efficiency, easy available with minimum side effects [3, 13]. This led the researchers to find new antimicrobial agents from traditionally used medicinal plants.

In most of the developing countries including Ethiopia medicinal plants are used as a first medicinal response in many diseases including bacterial infection diseases [14]. Ethiopia has a superb traditional health care system based on traditionally used medicinal plants [15, 16]. However, many studies have so far been concentrated in some parts of the country [17, 18], and there exist knowledge gap due to the absence of documentation [19] particularly, in *Itang* Special District, *Gambella* Region, Ethiopia. Even though, the communities of this area have been relying on a number of medicinal plants for treatment of various ailments, there is no scientific document on medicinal plants. Tamarind (*Tamarindus indica*), a mono-species leguminous tree of the family *Fabaceae* [20] is a pan-tropical species native to tropical Africa including Ethiopia [21, 22], and is often a multipurpose plant used either nutritionally [22], or medicinally [23–25], and is widespread in *Itang* Special District where it mostly grows wild.

Although antibacterial activity of ethanol and aqueous extract of *T. indica* fruit pulp against some bacterial strains using agar well diffusion and macro broth dilution techniques have been reported in Nigeria [26]. However, the antibacterial activity of medicinal plant extracts dependent on various factors; namely the solvent that used for the extraction [27], the environmental and climate conditions under which the plant grew could affect the accumulation of bioactive pharmaceutical ingredients that are found in the plants [28], test concentration of the extract, the choice of the extraction method, the method of antibacterial activity test and the test microorganisms [29]. Disc diffusion assay offers many advantages over other methods: simplicity, low cost, the ability to test enormous numbers of microorganisms and antimicrobial agents, and the ease to interpret results [30].

Before this study, evidence on *Tamarindus indica* as medicinal plant was purely anecdotal for the native communities of Gambella, Ethiopia as the plant is cherished only for the nutritional values of the fruits. Therefore, it is selected for this study due to the claims by some of the natives as having medicinal values, and was designed to addresses a neglected aspect by investigating the effect of fruit pulp crude extracts on the selected pathogenic bacteria, with an intention to add new knowledge to the communities’ traditional use of plants, and a stepping stone for further researches on novel plant-based therapies. Therefore, the present study was carried out to investigate the in vitro antibacterial activity of *Tamarindus indica* fruit pulp extracts using disc diffusion technique against some selected pathogenic bacteria.

## Materials and methods

### Sample collection and preparation

Ripe (dried) fruits were collected from Pulkot kebele (small administrative unit) (Latitude: 8°5′N and Longitude: 33°55′E) found in *Itang* Special District, Gambella Region, 35 km away from Gambella city and 801 km away from Addis Ababa, Ethiopia in January, 2022. The sample was collected from private land up on the verbal consent obtained from the land owner. Pulkot kebele is flat terrain and classified as lowland with the altitude of about 380 m a.s.l. The annual temperature has a minimum and maximum of 18.09 °C and 39.34 °C respectively, while the rainy season having annual average rain fall of 1500–2000 mm.

These pods (fruits) were cut by scissors, wrapped in newspapers and put in a sealable plastic bag. The sample authentication was carried out and the identification of the plant material based on the morphological criteria by botanist (Tariku Berhun). The authentication was confirmed at the National Herbarium, Addis Ababa University. Specimen was kept in Addis Ababa University Herbarium with number: G.G.1/22. The fresh fruits of *T. indica* were rinsed thoroughly using tap water, and chopped to tiny pieces and the pulp covering the seeds and the shell was removed using hand-scrapped and air dried at room temperature (23 ± 2 °C) for 15 days with careful and constant follow up to avoid any contamination. Then it was ground using a grinder and then sieved (0.5 mm mesh), to obtain an appropriate and uniform particle size. The sample was labeled and stored in closed glass bottles at − 20 °C until used for further analysis.

### Sample extraction

Subsequent extraction method was employed to get crude extracts using two analytical grade solvents with increasing polarity acetone obtained from Loba Chemie Pvt. Ltd.), and ethanol obtained from Alpha Chemika, (India). Maceration technique was chosen for obtaining the extract due to its excellent efficiency, and by adopting the protocol described by Geremew Tafesse et al. [31] with minor modification. The crude extraction was carried out starting with acetone, and followed by ethanol. One hundred g of fruit pulp powder was macerated for 24 hr. in acetone with the ratio of 1:5 (w/v). Then after, the extract was filtered using whatman no.1 filter paper giving filtrates and residue. The residue was then macerated in ethanol for another 24 hr. with similar ratio. The filtrates were evaporated to dryness under vacuum at 45 °C using a rotary evaporator (Buchi, 300 series, Switzerland). The extraction was done in triplicate and the obtained crude mass was weighed in grams (g), and stored in small sealed plastic bottle container at − 20 °C until used for further investigation.

### Antibacterial activity

#### Test bacteria

Two bacterial species were used in this study, namely *K. pneumoniae* (ATCC 700603) and *S. aureus* (ATCC 25923), on the basis of their pathogenicity to cause frequent and serious infections in humans. These strains were supplied by the Ethiopian Biodiversity Institute (EBI), Addis Ababa, Ethiopia.

#### Preparation of test solutions

The crude extracts were diluted to make three different concentrations of 100, 200 and 300 mg/mL as working stock solutions according to Bahiru M. et al. [32]. The first working solution was prepared by transferring 100 mg of each extract to sterile 1 mL test tube containing some amount of 3% Tween 20. The solution is diluted to the mark to obtain a concentration of 100 mg/mL. The second and the third working concentrations were prepared by similar manner. The prepared stock solutions were stored at − 20 °C until used.

#### Antibacterial tests

The antimicrobial activity (antibacterial sensitivity test) of the plant extract was done using the disk diffusion method according by Tafesse G. et al. [31], and Ismael J. et al. [27]. Paper disks were punched out from a sheet of absorbent filter paper by an ordinary office two-hole puncher. These were dispensed in batches in screw-capped bottles and sterilized at 121 °C for 1 hr. The two bacterial strains were activated on their selective media: MacConkey agar for *K. pneumoniae* and Mannitol-Salt agar for *S. aureus*, and were incubated at 37 °C for 24 hr.

Few colonies of each strain were transferred with a sterile inoculating loop to a nutrient broth until turbidity is adjusted to that of McFarland 0.5 turbidity standard. The plates containing Muller-Hinton agar were prepared where the two bacterial strains were streaked using sterile cotton swabs. The external surface of each plate was divided into five parts as each confine five paper discs: three discs containing extracts at different concentrations, one for the positive control and the remainder as negative control.

The disks were loaded with 50 μL of the crude extract from each of the three measured concentration, at a separate quadrant of each plate. One disk containing Tetracycline at 2.5 mg/mL, and a disk immersed in 1 mL of 3% Tween 20, each was kept as positive and negative controls respectively. These plates were then incubated at 37 °C for 24 hr. after which, the diameter of the zone of inhibition (ZI) was measured in millimeter (mm), and the tests were done in triplicate.

#### Determination of minimum inhibitory concentrations (MIC)

The MIC of crude extracts was determined according to method described by Tafesse G. et al. [31], and Ismael J. et al. [27]. The disk diffusion method was employed as in the susceptibility tests, except that the disks were immersed in each prepared concentration of the samples, and triplicate tests were performed at 300, 150, 75, 37.5, 18.75, 9.38 and 4.69 mg/mL.

#### Data analysis

All the data obtained from the experimental result were recorded by measuring (in mm) zones of growth inhibition (ZI) by the controls and of each crude extract on each bacterium, then taking the average (Mean ± standard error of the mean (SEM)) value of three tests. The results were compared by using one-way analysis of variance (ANOVA)/Tukey’s Honesty Significant Difference (HSD) test, with 95% confidence intervals (CI) where *P*-value is less than 0.05 showing significant difference.

## Results and discussions

### Antibacterial activity

The extract using intermediary polar solvent (acetone) and a more polar solvent (ethanol), subsequent extraction resulted in two crude extracts of *T. indica.* The result revealed that the fruit pulp acetone extract ethanol extract were 9.1 g (9%) and 19.5 g (24%) respectively. Obviously, a higher percentage yield was obtained using ethanol solvent, which is consistent with the previous study by Bahiru M. et al. [32]. The possible explanation for this might be reflecting the polarity of the solvent. Hence, the ethanol is very effective to extract polar compounds due to its high polarity and good solubility for polar compounds [33]. However, Nwodo UU, et al. [26] obtained small amounts of ethanol fruit pulp extract yield, in contrast to the current study. The difference may be due to the difference in environment and climate condition, season of the plant collection, and growth stage of the plant [28, 29].

The *fruit pulp* crude extracts of the *T. indica* exhibited antibacterial activity against the tested bacteria strains (Table 1). According to the results obtained from the disc diffusion assay (Table 1), the acetone crude extract of the fruit pulp showed effectiveness against *S. aureus*, with the three set concentrations, 100, 200 and 300 mg/mL of the extract showing respective mean growth inhibition of 13.00 ± 0.57, 16.33 ± 0.33 and 17.00 ± 0.57 mm while the positive control at concentration of 25 μg/mL had an average growth inhibition of 15.57 ± 0.033 mm (Table 2). The obtained result revealed that there is no statistically significant difference between the mean inhibition scores of these extracts concentrations and the positive control (drug) (*P* > 0.05). However, there is significant difference between the mean inhibition scores of these extract at all test concentrations with the 3% Tween 20 (negative control) (*P* = 0.00) (Table 2). The significance difference between the crude extract with that of the negative control and the positive control clearly illustrate that the *T. indica* fruit pulp crude extract has an antibacterial activity towards the test organism (*S .aureus*).

**Table 1:** Growth inhibitory level of fruit pulp extracts of *T. indica* against pathogenic bacteria as compared to the tetracycline antibiotic (positive control) and Tween 20 (negative control)

Extract	Concentration (mg/mL)	Effect level	
		Tested bacteria	
		*S. aureus*	*K. pneumonia*
Tween 20	1	–	–
Tetracycline	0.025	++++	++++
Acetone	100	+	++
	200	+++	+++
	300	++++	++++
Ethanol	100	++	++
	200	+++	+++
	300	++++	++++

Key: *S. aureus (Staphylococcus aureus (ATCC 25923)), K. pneumonia (Klebseilla pneumonia (ATCC 700603))*; ˗, +, ++, +++, and ++++ shows the extract used could have no effect, weak effect, moderate effect, strong effect, and very strong effect on the growth of the selected organisms respectively

**Table 2:** Zone of inhibitory activity of the fruit pulp extracts of *T. indica* against pathogenic bacteria as compared to the tetracycline drug (positive control) and Tween 20 (negative control)

Extract	Concentration (mg/mL)	Diameter zone of inhibition (ZI) (mm)			
		Tested bacteria			
		*S. aureus*		*K. pneumonia*	
		Mean ± SD	P-value	Mean ± SD	*P*-value
Tetracycline	0.025	16.00 ± 0.33^a^		16.00 ± 0.57^a^	
Acetone	100	13.00 ± 0.57^b^	0.00	14.33 ± 0.67^a^	0.81
	200	16.33 ± 0.33^a^	0.79	17.33 ± 1.40^a^	0.90
	300	17.00 ± 0.57^a^	0.24	18.67 ± 1.76^a^	0.46
Tetracycline	0.025	16.00 ± 0.57^a^		16.00 ± 0.33^a^	
Ethanol	100	13.33 ± 0.88^a^	0.24	13.67 ± 0.33^b^	0.00
	200	14. 67 ± 1.20^a^	0.79	15.33 ± 0.33^a^	0.06
	300	16.00 ± 1.00^a^	1.00	17.67 ± 0.33^a^	0.20

Key: *S. aureus (Staphylococcus aureus (ATCC 25923)), K. pneumonia (Klebseilla pneumonia (ATCC 700603))*; ˗, +, ++, +++, and ++++ shows the extract used could have no effect, weak effect, moderate effect, strong effect, and very strong effect on the growth of the selected organisms respectively. Mean values with different superscripts in the same column are significantly different

Similar evidence also happened against *K. pneumoniae* when tested on the fruit pulp crude acetone extract. The mean score of inhibition for extract at concentrations of 100, 200 and 300 mg/mL were 14.33 ± 0.67, 17.33 ± 1.40 and 18.67 ± 1.76 mm respectively. The obtained result were compared with the drug (positive control) at concentration of 25 μg/mL and the mean of inhibition was 16.00 ± 0.57 mm, the respective *p*-values were (*P* = 0.81, 0.90 and 0.46). These clearly indicate that, there is no significant difference between the inhibition of the *T. indica* fruit pulp crude extract at all tested concentrations with that of the drug (*P* > 0.05) (Table 2), nevertheless, there were significant differences with the negative control (*P* = 0.00) at all the test concentrations.

The fruit pulp ethanol extract revealed the effectiveness of the extract on both tested bacteria strains (*S. aureus* and *K. pneumoniae*) and the result was presented as shown in Table 1. The fruit pulp ethanol extract at 100, 200 and 300 mg/mL concentrations showed average mean inhibition zone of 13.33 ± 0.88, 14.67 ± 1.20 and 16.00 ± 1.00 mm, respectively, against *S. aureus.* The antibacterial activity result for each concentration of the extract were compared with the positive control (25 μg/mL of Tetracycline) whose mean inhibition score is 16.00 ± 0.57 mm, the respective *p*-values were (*P* = 0.24, 0.79 and 1.00). These clearly indicated that, there is no significant difference between the inhibition zone of the extract at all tested concentrations with that of the drug (*P* > 0.05) (Table 2). However, the obtained result at all tested concentrations were significantly different with the negative control (*P* = 0.00). The obtained result confirms that the antibacterial activity of the *T. indica* fruit pulp ethanol extract of against the tested bacterium (*S. aureus*). Similarly, results of the fruit pulp ethanol extract, against *K. pneumoniae* at concentrations of 100, 200 and 300 mg/mL showed the mean inhibition zones of 13.67 ± 0.33, 15.33 ± 0.33 and 17.67 ± 0.33 mm, respectively (Table 1). However, statistically, no significant difference was found between the inhibition zones of the ethanol extract at all concentrations with that of the drug (positive control) (*P* > 0.05) (Table 2), while at all concentrations the result showed significant difference with that of the 3% Tween 20 (negative control) (*P* = 0.00). This also explains the antibacterial activity of the crude extract against the given tested bacterium (*K. pneumoniae*).

The present study clearly demonstrates the sequentially acetone and ethanol fruit pulp extract of *T. indica* showed a remarkable antibacterial activity against both Gram-positive and Gram-negative bacterial species [26, 27]. This result further enriches the pharmaceutical value of the neglected fruit pulp of *T. indica* and also confirms the validity of the traditional uses of *T. indica*. Even though most of the previous studies were done on the antibacterial activities of medicinal plants [27, 34–39], however, the present study confirmed the bioactivity of the fruit pulp of *T. indica* by showing higher antibacterial activity, thus authenticating the medicinal value of the fruit pulp of *T. indica*. This could be as a result of the secondary metabolite particularly terpenoids’ family had antibacterial activity and able to penetrate the bacteria cell wall which may be due to the lipophilic nature of the terpenoids’ family [2, 26, 33]. Hence, the antibacterial activities of plant extracts may be linked with the presence of secondary metabolites, which play important role in the treatment of bacterial infections [40–42]. Thus fruit pulp extract of *T. indica* can be used as alternative medicines for bacterial infections [26].

### Determination of minimum inhibition concentration (MIC)

Interestingly, this study found that, both acetone and ethanol extracts from the fruit pulp of *T. indica* showed antibacterial activities, and that dilutions of various concentrations can inhibit the growth of the investigated bacteria strains, (*S. aureus* and *K. pneumoniae*) (Table 3). Accordingly, *K. pneumoniae* strain showed more sensitivity to both the acetone and ethanol fruit pulp extracts compared to *S. aureus* strain, with the respective minimum inhibitory concentration (MIC) values recorded at 9.38 mg/mL for the former and 18.75 mg/mL for the later bacterial strain (Table 3). These results indicate that fruit pulp has antibacterial activity as revealed in the sensitivity of the tested bacteria strains (*S. aureus* and *K. pneumoniae*) towards the fruit pulp extracts of *T. indica*.

**Table 3:** Minimum inhibition concentration (MIC) of fruit pulp extracts of *T. indica*

Extract	Concentration (mg/mL)	Activity	
		Tested bacteria	
		*S. aureus*	*K. pneumonia*
	4.6875	–	–
Acetone	9.375	–	**+**
	18.75	**+**	**+**
	37.5	**++**	**++**
	75	**++**	**+++**
	150	**+++**	**+++**
	300	**++++**	**++++**
	4.6875	**–**	**–**
Ethanol	9.375	**–**	**+**
	18.75	**+**	**+**
	37.5	**+**	**++**
	75	**++**	**+++**
	150	**++**	**+++**
	300	**+++**	**++++**

Key: *S. aureus (Staphylococcus aureus (ATCC 25923)), K. pneumonia (Klebseilla pneumonia (ATCC 700603))*; ˗, +, ++, +++, and ++++ shows the extract used could have no effect, weak effect, moderate effect, strong effect, and very strong effect on the growth of the selected organisms respectively

The determination of MIC revealed that a different minimum concentration of the *T. indica* crude extract could inhabit the growth of the reference bacteria (*Gram-negative strain (K. pneumoniae (ATCC 700603)) and Gram-positive strain (S. aureus (ATCC 25923))*. The least MIC value was recorded for *Gram-negative strain (K. pneumoniae (ATCC 700603) for* both solvent extracts. This could explain that *Gram-negative bacterial strain had more susceptible to T. indica* fruit pulp crude extract*.*

Comparison of the findings with those of other studies confirms, this outcome broadly backings the work of other studies in this area involving the fruit pulp extract against pathogenic bacteria. To mention a few, Das and Banerjee [24] reported the antibacterial activity of methanol extract on *Bacillus subtilis;* study by Abdallah and Muhammad [43] on the leaf and fruit pulp against *E. coli* and *Shigella* sp. showed that both aqueous and methanol extracts have antibacterial properties, and the work of Dorcas et al. [44], which, also reported that the aqueous and alcoholic (ethanol, methanol and isopropanol) extracts were highly susceptible on *E. coli, Bacillus* sp.*, Staphylococcus* sp.*, Klebsiella* sp. and *Pseudomonas* sp., all of which were credited to the presence of flavonoids, alkaloids, saponins.

Similarly, a more recent study by Fagbemi et al. [45] on bioactive compounds, antibacterial and antioxidant activities of methanol extract against *Escherichia coli*, *Klebsiella pneumoniae*, *Pseudomonas aeruginosa*, *Acinetobacter calcaoceuticus*, *Plesiomonas shigelloides Bacillus cereus*, and *Staphylococcus aureus*, also revealed the effectiveness of the extract on all the tested bacteria strains, and gave credit to the presence of β-sitosterol, cis-Vaccenic acid and other compounds like alkaloids and flavonoid as reported by other studies. The present study therefore, confirms previous findings and backs the evidence that suggests the involvement of fruit pulp in pharmaceutical products against pathogenic bacteria.

Prior studies have noted the importance of *T. indica* in traditional medicine, for well-known health benefits including wound healing, snake bite, abdominal pain, colds, inflammations, diarrhea, helminth infections and fever [46], other reports have also shown its role as antimicrobial, antidiabetic, anti-inflammatory probably due to the presence of a variety of bioactive compounds in many parts which can be used as an indicator on the possibility of the *T. indica* fruit pulp extract application in the pharmaceutical industry [25, 47].

Though both acetone and ethanol extracts showed high level of effectiveness on the test bacteria, *K. pneumoniae* showed more sensitivity to the extract than *S. aureus* (Table 1). The study results confirmed that there were impressive in confirming the antibacterial activity. Thus this study result validates the traditional application of the plant under study along with suggesting the possibility of developing drugs from it. Although the findings should be interpreted with caution, this study has several strengths: it enhances the researches that highlight the potential usefulness of the plant in having antimicrobial properties, it will be of use to the scientific and biomedical communities by paving the way for other researchers, and promote community awareness in conserving the plant not only as a source of food but also as a medicinal treasury.

## Conclusion

The outcome of this study suggests that both the acetone and ethanol fruit pulp extracts of *T. indica* showed strong antibacterial activity as it showed inhibiting the growth of tested pathogenic bacteria similar to that of the drug (Tetracycline). Generally, acetone and ethanol extracts showed the highest antibacterial activity against the Gram-negative bacterial strains (*K. pneumoniae (ATCC 700603)*) than Gram-positive bacterial strains *(S. aureus (ATCC 25923))*. The study revealed that *T. indica* contain considerable amount of compounds responsible for antimicrobial activities, which can be used as easily accessible source for the pharmaceutical applications. However, further studies are necessary on the isolation and characterization of individual compounds to elucidate their different antimicrobial mechanisms. The isolated compounds need to be evaluated in scientific manner using scientific animal models and clinical mechanisms of action in search of bioactive molecules. This research has thrown up questions in need of further investigation on other bacteria as well as on its antifungal activity.

## Data Availability

The datasets used and/or analyzed during the current study available from the corresponding author on reasonable request.
